# Effects of beinaglutide on visceral fat area and gut microbiota in obesity

**DOI:** 10.1186/s40001-025-02585-5

**Published:** 2025-06-04

**Authors:** Tianshui Liu, Weizheng Liang, Fanghua Xu, Yun Wu, Jun Su, Chaoxi Li, Weidong Ren, Li Shi

**Affiliations:** 1 Graduate School, Hebei North University, Zhangjiakou, China; 2 Central Laboratory, The First Affiliated Hospital of Hebei North University, Zhangjiakou, China; 3 Nutrition Section, The First Affiliated Hospital of Hebei North University, Zhangjiakou, China; 4 Department of Ultrasound Medicine, The First Affiliated Hospital of Hebei North University, Zhangjiakou, China; 5 Department of Endocrinology, The First Affiliated Hospital of Hebei North University, Zhangjiakou, China; 6 International Medical Services Department, The First Affiliated Hospital of Hebei North University, Zhangjiakou, China

**Keywords:** Obesity, Beinaglutide, GLP-1RA, Gut microbiota, Visceral fat area

## Abstract

**Background:**

The gut microbiota plays a substantial role in obesity. Glucagon-like peptide-1 receptor agonists (GLP-1RAs) can affect body weight, insulin resistance, and lipid metabolism via combined hypothalamic feeding centre and gastrointestinal tract activity. It also affects the intestinal flora of patients with obesity. We investigated changes in visceral fat and gut microbiota of patients with obesity treated with beinaglutide.

**Methods:**

Thirty-three patients from our hospital with obesity treated with beinaglutide and 20 sex- and age-matched healthy controls were included based on the inclusion and exclusion criteria. Glycolipid metabolism indices, inflammation indices, and hepatic and renal functions were assessed at treatment initiation and 4 and 12 weeks after treatment. Body weight, waist circumference, blood pressure, and body composition (visceral fat area, body fat, body fat percentage, skeletal muscle, and basal metabolic rate) were measured. The Homeostatic Model Assessment of Insulin Resistance index was calculated. Fecal samples were collected from 12 cases. Effects on intestinal flora were assessed by sequencing the V3–V4 region of the 16S rRNA gene.

**Results:**

Beinaglutide significantly reduced visceral fat area, improved glycolipid metabolism, and decreased total cholesterol, low-density lipoprotein cholesterol, blood glucose, and C-reactive protein levels. It also considerably restored the α-diversity of intestinal flora, with decreased in relative abundances of *Prevotella*, *Lachnospira and Dialister* at the genus level and increased in the relative abundance of *Blautia A* and *Gemmiger A*.

**Conclusions:**

Our findings provide insights into the mechanisms by which beinaglutide aids weight loss, particularly via modulating the gut microbiota.

**Graphical abstract:**

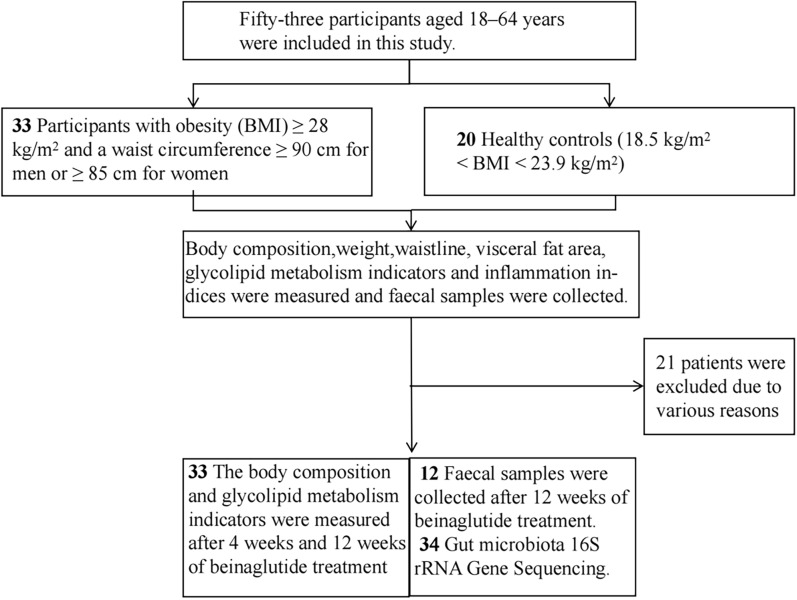

**Supplementary Information:**

The online version contains supplementary material available at 10.1186/s40001-025-02585-5.

## Introduction

Obesity is a global public health concern, and the combined prevalence of adults categorised as being overweight and obese in China is expected to reach 65.3% in 2030 [1]. Abdominal obesity is often accompanied by an increase in visceral fat and associated with metabolic syndromes, including dyslipidaemia, diabetes mellitus, and hypertension [2, 3]. The gut microbiota plays a crucial role in the development of obesity [4]. The intestinal microbiota can alter GLP-1 secretion by the intestinal epithelium, thus regulating gastrointestinal motility and metabolism [5].

**Fig. 1:**
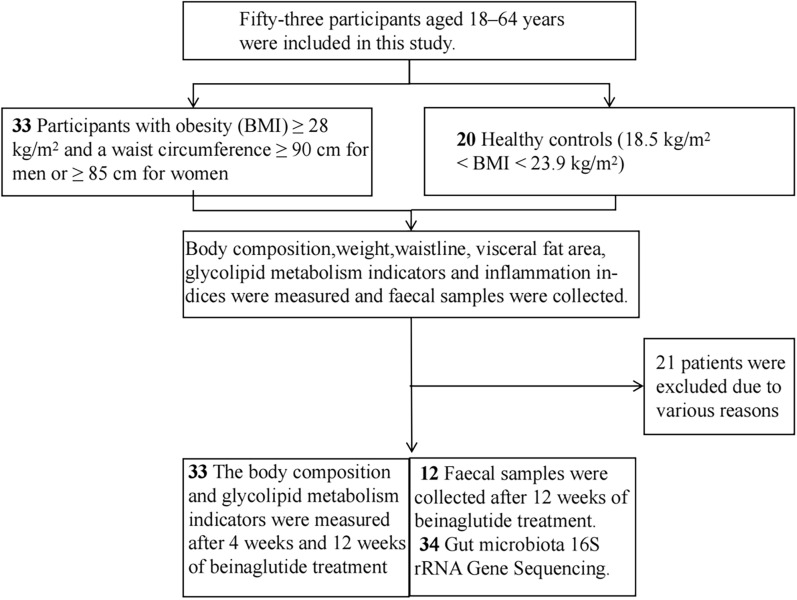
Flow chart of this study

Beinaglutide is a glucagon-like peptide-1 receptor agonist (GLP-1RA) that mimics the physiological action of GLP-1. Through its effects on the hypothalamic feeding centre and the gastrointestinal tract, beinaglutide can be used to reduce food intake by suppressing appetite and delaying gastric emptying, particularly when administered before meals. As such, it has a positive effect on body weight, insulin resistance, lipid metabolism, and inflammation [6, 7]. Beinaglutide also increases the relative abundance of *Faecalibacterium prausnitzii* in the gut of patients with type 2 diabetes mellitus [8]. In addition, an increased relative abundance of *F. prausnitzii* can stimulate basal metabolic activity and improve energy metabolism, which in turn lead to weight loss [9]. Both liraglutide and semaglutide reduced body weight in obese experimental animals via modulating intestinal flora structure [10, 11]. However, to the best of our knowledge, studies on the gut microbiota of patients treated with beinaglutide are lacking. Herein, we characterised the changes in visceral fat area and intestinal flora of patients with obesity after beinaglutide treatment (Fig. 1).

## Materials and methods

### Study design and participants

Fifty-three participants aged 18–64 years were included in this study. Thirty-three of the participants were individuals with obesity seeking treatment at the weight loss clinic of the First Hospital Affiliated to Hebei North College, Zhangjiakou, Hebei province, China from August 2023 to July 2024. According to the 2022 Expert Consensus on Obesity Prevention and Control for Chinese Residents, the inclusion criteria were a body mass index (BMI) ≥ 28 kg/m^2^ and a waist circumference ≥ 90 cm for men or ≥ 85 cm for women. The exclusion criteria were obesity secondary to cortisolism, hypothyroidism, hypothalamic, or pituitary disorders and genetic mutations; pregnancy or breastfeeding; hepatic or renal dysfunction; history of heart failure, stroke, or ischaemic heart disease; severe neurologic or psychiatric disorders; malignant neoplasms; alcohol or drug abuse. Twenty sex- and age-matched participants who received health checkups at the Health Management Center and had a healthy BMI (18.5 kg/m^2^ < BMI < 23.9 kg/m^2^) were selected as controls. After 12 weeks of unsuccessful lifestyle-guided weight loss, the participants with obesity were subcutaneously treated with beinaglutide (0.06 mg) 5 min before each meal, three times a day. The dose was gradually increased to 0.1 mg within 2 weeks based on clinical response and maintained at until the end of the study period. Patients underwent regular follow-ups, including biochemical assessment and body composition measurements after 4 and 12 weeks, respectively (data were compared with initial measurements before treatment). All participants were informed that no other GLP-1RAs could be used. The medical dietitian developed an energy-restricted balanced diet for each participant during their initial examination, with caloric restriction set at the basal metabolic rate of patients with obesity minus 500 kcal. This was designed to meet daily energy requirements with a carbohydrate content of 45–60%, lipid content of 20–30%, and protein content of 10–20%. Fibre intake was 14 g/1000 kcal, with 7–13 g soluble fibre. Moderate-intensity aerobic exercise (minimum of 30 min) combined with resistance exercise (minimum of 15 min) was conducted 3–5 times per week. Twenty-four faecal samples were collected from the obese population both before and after beinaglutide use, and 10 faecal samples were collected from the healthy population; some patients were excluded due to various reasons, such as the use of antibiotics. The 34 faecal samples were then analysed using 16S rRNA gene sequencing to assess composition and diversity of the intestinal microbiome. The primary endpoints were changes in the visceral fat area and gut flora. Secondary endpoints included BMI, waist circumference, blood pressure, body composition, body fat, body fat percentage, skeletal muscle, glucose-fat metabolism, Homeostatic Model Assessment of Insulin Resistance (HOMA-IR), inflammatory markers, hepatic and renal function, as well as the occurrence of adverse events. Adverse events were recorded during the trial and up to 14 d after the treatment. Serious adverse events were immediately reported to the research ethics committee of the study hospital and to the Institutional Review Board of the institutional office for drug clinical trials. Each participant provided signed informed consent prior to the start of the study. This prospective study was reviewed and approved by the Ethics Committee of the First Hospital Affiliated to Hebei North College (July 25, 2023, No. K2024149).

### Clinical data and body composition determination

The sociodemographic characteristics of participants, including sex and age, were recorded Blood pressure was measured using a standard sphygmomanometer. All participants were instructed to record their body composition measured using a body composition analyzer (BioSpace InBody S10, Korea), especially visceral fat area (cm^2^), and basal metabolic rate was determined using a lung function tester (EXPRESS CCM, McAfee Inc.) after 8–10 h of fasting and following the voiding of urine and faeces.

### Biochemical indices

After an overnight fast of 8–10 h, blood samples were collected from the elbow veins of patients in the morning before treatment initiation and during beinaglutide treatment. The samples were analysed for triglycerides (TG), total cholesterol (TC), alanine aminotransferase (ALT), aspartate aminotransferase (AST), triglycerides (TG), cholesterol (TC), low-density lipoprotein cholesterol (LDL-C), high-density lipoprotein cholesterol (HDL-C), γ-glutamyl transferase (GGT), alkaline phosphatase (ALP), fasting glucose (FPG), uric acid (UA), urea, Cr (creatinine), and hypersensitive C-reactive protein (CRP) via high-performance liquid chromatography using a fully automated biochemical analyser Automatic Analyzer 7600 (HITACH, Tokyo, Japan) in the hospital laboratory. Glycated haemoglobin A1c (HbA1c) was also measured using an immunoassay. Fasting insulin (FINS) levels were measured using a chemiluminescence immunoassay. Adiponectin (ADPN) was measured using latex-enhanced immunoturbidimetric assay. The steady-state model assessment of insulin resistance was calculated as follows: insulin resistance = [fasting glucose (mmol/L) × FINS (µIU/mL)]/22.5. The estimated glomerular filtration rate was determined using the Chronic Kidney Disease Epidemiology Collaboration (simple CKD–EPI formula) [12].

### Faecal DNA extraction, 16S rRNA gene sequencing, and data analysis

Early morning fasted faecal samples (from the middle section) from healthy controls and participants with obesity were collected in sterile stool tubes before and after 12 weeks of drug administration. The fresh stools were placed in an ice box and transferred to a designated staff member within 1 h. The faecal samples were consecutively numbered and stored at − 80 °C. Faecal DNA was extracted using the HiPure Stool DNA Kit (Beijing Tiangen Biochemical Technology Co. China) according to the manufacturer’s instructions. In the experiment, human fecal DNA was extracted and operated in strict accordance with the instructions of the kit. 220 mg of frozen feces was first placed in a centrifuge tube, and grinding beads, aromatic sulfonic acid buffer, sodium hydrochloride citrate buffer, and proteinase K were successively added to fully mix the mixture. Ribonuclease A degraded the RNA, and the synergistic effect of the lysis solution and ribonuclease was utilized to fully cleave the RNA and remove the impurities, and the temperature and time were strictly controlled in the process. During the process, the temperature and time were strictly controlled to ensure the thoroughness of the cleavage without damaging the DNA, and then the mixed solution was centrifuged, washed and adsorbed for several times to finally isolate and purify the required bacterial DNA. The concentration and integrity of the DNA were quality checked using a NanoDrop microspectrophotometer SpectraMax® QuickDrop™ (Molecular Devices, Silicon Valley, CA, USA) and agarose gel electrophoresis. The V3 + V4 region of *16S* rDNA was amplified using specific primers and barcodes. The primer sequences were as follows: 338F, 5′ ACTCCTACGGGAGGCAGCA 3′ and 806R, 5′ GGACTACHVGGGTWTCTAAT 3′. The *16S* amplification and sequencing of the above DNA were completed using an Illumina platform (San Diego, CA, USA). Sequencing and data analysis services were provided by Wuhan Bioinformatics CO. Ltd. (Hubei province, China). The data were analysed to obtain the Effective Tag, based on which operational taxonomic unit abundance statistics, species annotation, and biodiversity analyses were performed.

### Statistical analysis

Statistical analyses were conducted using IBM SPSS version 26.0 (IBM, Armonk, NY, USA). Quantitative data were described using means, standard deviations, and medians, whereas qualitative data were described using numbers and percentages. Quantitative data were tested for normality using the Shapiro–Wilk test, and parametric tests were used if the data were normally distributed. Nonparametric tests were used if the data were skewed. Independent *t* tests or Mann–Whitney *U* tests were used to compare two independent groups of samples. Two or three variable observations were compared using the paired-samples *t* test or repeated one-way ANOVA for normally distributed data and the Wilcoxon test or Friedman's test for non-normally distributed data. Statistical significance was assumed at *p* < 0.05.

## Results

### Baseline characteristics and pre-weight loss clinical characteristics of participants

A total of 53 participants met the study criteria, including 33 patients classified as obese and 20 healthy individuals who were used as controls, with mean ages of 35.33 ± 8.00 and 32.95 ± 7.43, respectively. The baseline characteristics and clinical outcomes of patients with obesity and healthy controls are presented in Table 1. Comparison of baseline information between the two populations using the independent sample *t* tests, Mann–Whitney, and chi-square tests. There were no statistical differences in data with respect to gender and age. Compared with healthy controls, patients with obesity showed significant increases in BMI, waist circumference, visceral fat area, body fat and body fat percentage (all *p* < 0.001). Heart rate and basal metabolic rate were significantly higher in patients with obesity than in the healthy population (*p* = 0.035, *p* = 0.045), and their skeletal muscle content was significantly increased (*p* = 0.011). TC, LDL-C, TG, blood glucose, and HbA1c increased to varying degrees in patients with obesity (*p* < 0.001,* p* = 0.001, *p* = 0.001,* p* < 0.001, and* p* = 0.001, respectively). UA levels were also significantly higher in patients with obesity than in healthy controls (*p* = 0.001). The levels of adiponectin, which is involved in glucolipid metabolism, were significantly lower in patients with obesity (*p* < 0.001). Patients with obesity tended to have more pronounced insulin resistance (*p* < 0.001) than healthy controls. Although fatty liver was observed on imaging ultrasound in patients with obesity, there were no significant differences in liver function indicators, ALT, AST, GGT, and ALP (*p* = 0.065, *p* = 0.985 *p* = 0.189, and *p* = 0.14, respectively), nor in indicators of renal function, including urea nitrogen, creatinine, and simple glomerular filtration rate (*p* = 0.659, *p* = 0.612, and *p* = 0.427, respectively). In conclusion, patients with obesity and healthy controls had significant differences in BMI, waist circumference, and other body composition parameters. In addition, patients with obesity exhibited significant dysregulation of glucose and lipid metabolism.

**Table 1:** Clinical characteristics of obese patients before and after the use of beinaglutide compared with the healthy controls

	Beinaglutide (33)			Control (20)	*P*	*P*_1_
	Baseline	4 weeks	12 weeks			
Sex (female/male)	25/8	–	–	12/8	0.138	–
Age (years)	35.33 ± 8.0	–	–	32.95 ± 7.43	0.285	–
WC (cm)	103.78 ± 7.18	95.95 ± 6.79a	89.68 ± 6.24ab	74.4 ± 10.29	< 0.001	< 0.001
BMI (kg/m^2^)	31.14 (30.34, 33.34)	28.97 (28.23, 30.58)a	27.50 (26.46, 28.90)ab	21.86 (20.32, 23.44)	< 0.001	< 0.001
HR (bpm)	81.61 ± 12.52	81.58 ± 9.6	80.97 ± 9.23	74.4 ± 10.23	0.035	0.963
SBP (mmHg)	136.48 ± 14.80	128.67 ± 12.66a	125.42 ± 11.18a	118.60 ± 8.73	< 0.001	< 0.001
DBP (mmHg)	91.82 ± 13.20	83.48 ± 11.00a	81.36 ± 9.77a	80.5 (70.5, 83)	< 0.001	< 0.001
TC (mmol/L)	4.80 (4.48,5.91)	4.49 (3.94,5.25)a	4.23 (3.64,4.84)a	3.97 ± 0.56	< 0.001	< 0.001
HDL-C (mmol/L)	1.12 (1.03,1.25)	1.03 (0.91,1.14)a	1.14 (1.03,1.26)b	1.20 ± 0.15	0.432	< 0.001
LDL-C (mmol/L)	3.00 ± 0.74	2.59 ± 0.54a	2.45 ± 0.63a	2.31 ± 0.60	0.001	< 0.001
TG (mmol/L)	1.7 (1.14,2.39)	1.27 (0.10,2.10)	1.06 (0.91,1.51)a	1.17 (0.84,1.38)	0.001	0.002
ALT (U/L)	33 (16,62.1)	27 (13,35)	18 (12.5,22.5)ab	22.5 (17.25,25.75)	0.065	< 0.001
AST (U/L)	23 (16,35)	21 (15,27.35)	17 (13,31)ab	23.5 (19.25,28.5)	0.985	< 0.001
GGT (U/L)	26 (18,37)	20 (14,26.5)a	19 (14.5,25)a	29.5 (23.75,32.5)	0.189	< 0.001
ALP (U/L)	71.68 ± 18.11	68.79 ± 18.48	69.26 ± 19.56	66.25 ± 7.95	0.14	0.405
UA (umol/L)	398 (305.5,433)	381 (320,453.5)	359 (284.5,416.9)b	303.72 ± 50.98	0.001	0.002
BUN (mmol/L)	4.37 ± 0.96	4.32 ± 0.94	4.48 ± 1.02	4.48 ± 0.66	0.659	0.518
Cr (umol/L)	56.78 ± 9.83	57.48 ± 8.30	57.27 ± 8.42	58.06 ± 6.73	0.612	0.498
eGFR (ml/min/1.73 m^2^)	117.80 ± 9.24	116.83 ± 9.33	117.37 ± 8.82	119.97 ± 10.1	0.427	0.222
ADPN (mg/L)	2.83 ± 0.70	–	3.55 ± 0.87	3.81 ± 0.99	< 0.001	< 0.001
HOMA-IR	4.18 ± 1.18	–	2.76 ± 0.56	1.16 (1.05,1.24)	< 0.001	< 0.001
FPG (mmol/L)	5.69 ± 0.52	5.51 ± 0.53	5.26 ± 0.49ab	5.01 ± 0.33	< 0.001	< 0.001
FINS (μIU/mL)	16.58 ± 4.59	–	11.93 ± 2.61	5.05 (4.96,5.54)	< 0.001	< 0.001
HbA1c (%)	5.5 (5.2,5.95)	–	–	5.2 (5.1,5.3)	0.001	–
hs-CRP (mg/L)	3.4 (1.8, 9.15)	1.9 (1.4, 6.25)a	2.2 (1.5, 4.95)a	0.5 (0.4,0.68)	< 0.001	< 0.001
Skeletal fat area (kg)	27.4 (26.05,32.75)	27.1 (25.3,31.55)a	27.2 (25.6,32.8)a	23.75 (21.5,33.08)	0.011	0.001
Body fat (kg)	33.90 ± 6.15	28.35 ± 5.18a	24.15 ± 5.26ab	14.81 ± 2.35	< 0.001	< 0.001
Percent body fat (%)	38.69 ± 5.82	35.44 ± 5.60a	31.34 ± 6.10ab	24.52 ± 5.4	< 0.001	< 0.001
VFA (cm^2^)	155.69 ± 29.60	127.84 ± 28.26a	103.38 ± 28.09ab	55.60 ± 19.88	< 0.001	< 0.001
BMR (kcal/day)	1644.88 ± 228.85	1566 ± 240.11	1549.43 ± 228.87a	1415.5 (1329,1739.5)	0.045	0.034
CAP (dB/m)	307 (285.5,328.75)	–	268 (255.5,285.75)	200.5 (184.75,235)	< 0.001	< 0.001
E (kPa)	7.45 (5.8,8.68)	–	5.55 (4.33,6.38)	5.08 ± 0.99	< 0.001	< 0.001
NAFLD n (%)	32 (96.97)	–	28 (84.85)	5 (15.1)	–	0.125
Hypertension n (%)	19 (57.58)	10 (30.30)	7 (21.21)a	–	–	< 0.001
Hyperlipidaemia n (%)	19 (57.58)	15 (45.45)	5 (15.15)b	–	–	0.001
Hyperuricaemia n (%)	13 (39.39)	11 (33.33)	8 (24.24)	–	–	0.18
IFG/IGT n (%)	7 (21.21)	3 (9.09)	1 (3.03)	–	–	0.031

### Pre- and post-weight loss clinical characteristics of participants

**Fig. 2:**
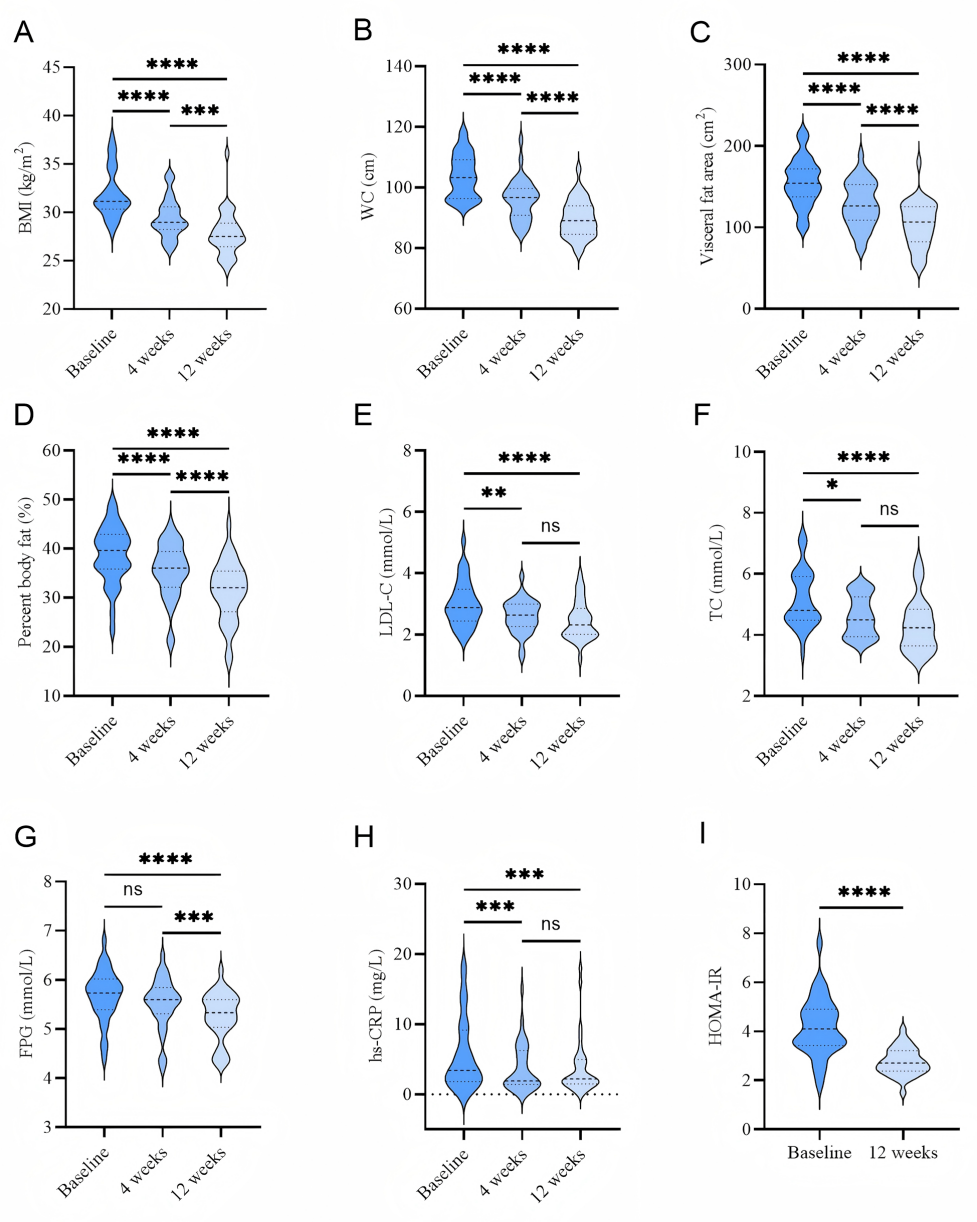
Clinical parameters of patients with obesity before and after beinaglutide treatment. **A**, **B**, **C**, **D** BMI, WC, visceral fat, and body fat percentage changes from baseline to 12 weeks. **E**, **F**, **G** LDL-C, TC and CRP changes from baseline to 12 weeks. **H** FPG changes from baseline to 12 weeks. **I** HOMA-IR changes from baseline to 12 weeks

Changes in the clinical indicators of patients with obesity after 4 and 12 weeks of beinaglutide treatment are shown in Table 1 and Fig. 2. The effect of beinaglutide on weight loss in patients was achieved within 4 weeks and persisted throughout the treatment period, showing significant decreases in BMI, waist circumference, visceral fat area, and body fat percentage compared with those at baseline (Table 1, Fig. 2A–D; *p* < 0.001, respectively). LDL-C, TC and inflammatory factor hs-CRP levels significantly decreased at week 4 (Table 1, Fig. 2E–G; *p* < 0.001). ANDP levels significantly increased after 12 week treatment (Table 1; *p* < 0.001), whereas FPG levels significantly decreased over time until week 12 (Table 1; Fig. 2H, p < 0.001). Furthermore, insulin resistance improved (Table 1, Fig. 2I; *p* < 0.001), while no significant changed in renal function was noted over time (Table 1; *p* = 0.222). Consistent with weight loss, we observed a decrease in basal metabolic rate at week 12 (Table 1; *p* = 0.034). It was pleasing to note that metabolic diseases associated with obesity such as hypertension, hyperlipidaemia and IFG/IGT were significantly improved in obese patients after 12 weeks of weight loss with beinaglutide, but hyperuricaemia and fatty liver did not improve significantly (Table 1) (*p* < 0.001, *p* = 0.001, *p* < 0.031, *p* = 0.18 *p* = 0.125).

### Relationship between changes in visceral fat area and obesity-related indicators

**Fig. 3:**
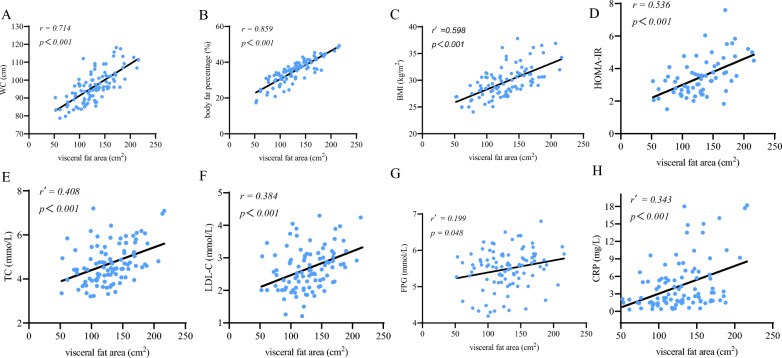
Relationship between visceral fat area and obesity-related parameters. **A** WC, **B** body fat percentage, **C** BMI, **D** HOMA-IR, **E** TC, **F** LDL-C, **G** FPG, and **H** CRP

Reducing visceral fat area reportedly improves insulin resistance. Patients with obesity receiving beinaglutide treatment exhibited a significant reduction in visceral fat area (*p* < 0.001). As expected, the reduction in visceral fat area was significantly and positively correlated with changes in waist circumference, body fat percentage and BMI (Fig. 3A–C), insulin resistance index, cholesterol, and LDL-C levels (Fig. 3D–F). However, there was no significant correlation with changes in glucose and CRP levels (Fig. 3G, H).

### Differences in gut flora between patients with obesity and healthy controls

**Fig. 4:**
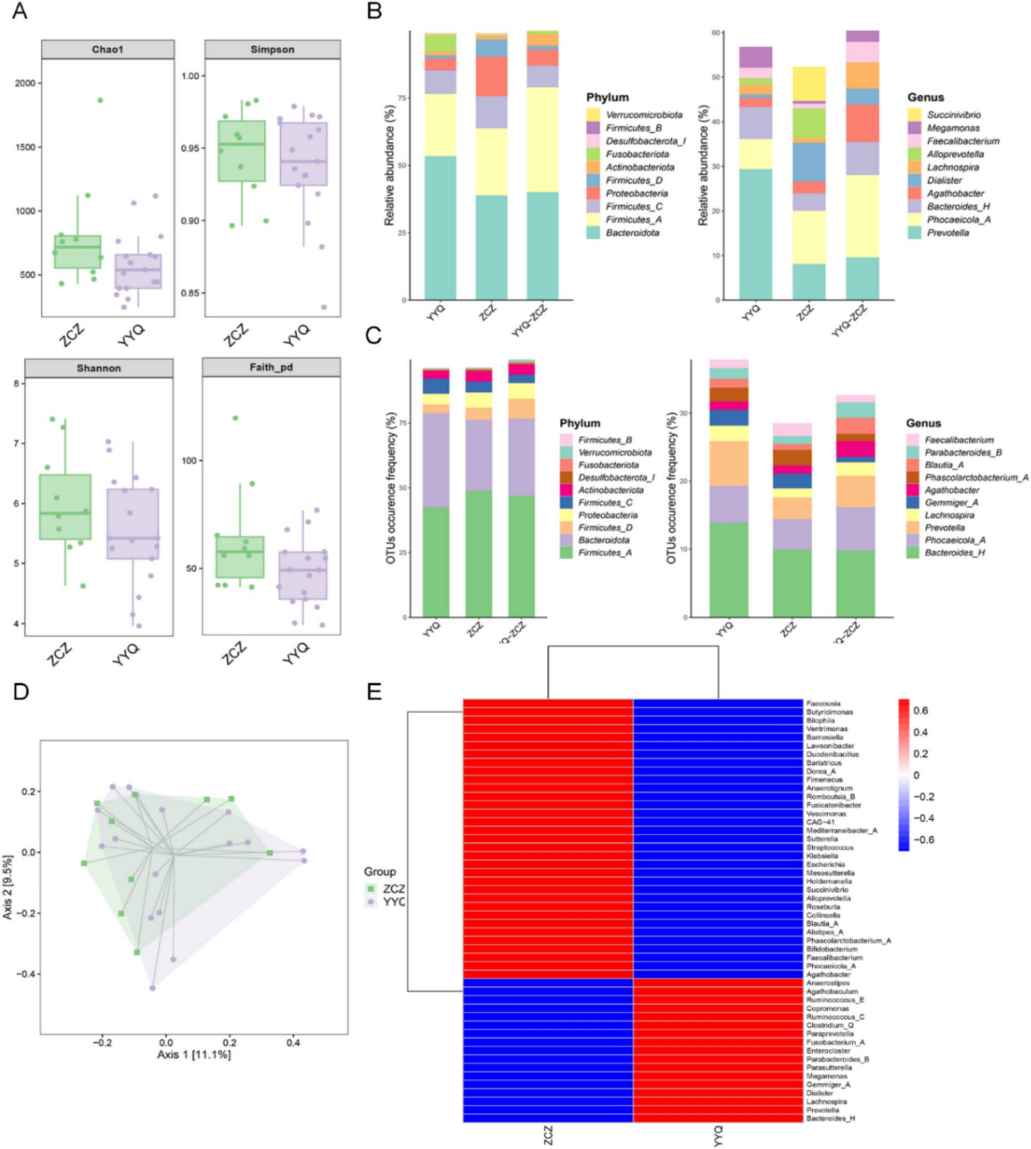
Differences in the gut microbiota of patients with obesity (YYQ) and healthy individuals (ZCZ). **A** α-Diversity of the gut microbiota (α-diversity indices: Chao1, Simpson, Shannon, and Faith PD indices). **B** Abundance of the corresponding amplicon sequence variant (ASV)/operational taxonomic unit (OTU) in each region at the phylum and genus level. **C** Number of corresponding ASVs/OTUs per region at the phylum and genus level. **D** β-Diversity of the gut microbiota **E** Heat map analysis of differences in intestinal flora between healthy individuals and patients with obesity

In both healthy individuals and patients before beinaglutide treatment, three specific phyla were consistently dominant: Firmicutes*,* Bacteroides, and Proteobacteria were the most abundant phyla in patients with obesity and healthy controls (Fig. 4B, C). α- and β-Diversity analyses were performed to determine between patients with obesity and healthy controls in gut microbiota diversity, revealing differences in the Chao1 and Faith PD indices (Fig. 4A, D). Overall, Genus-level analysis revealed a significantly higher relative abundance of *Prevotella*, *Anaerostipes*, *Parasutterella*, *Megamonas*, *Dialister*, and *Lachnospira* in patients with obesity than in healthy controls, while *Lawsonibacter*, *Roseburia*, *Collinsella*, *Blautia A*, *Phascolarctobacterium A*, *Bifidobacterium* and *Feacalibacterium* were significantly less abundant (Fig. 4E).

### Changes in intestinal flora after beinaglutide treatment in patients with obesity

**Fig. 5:**
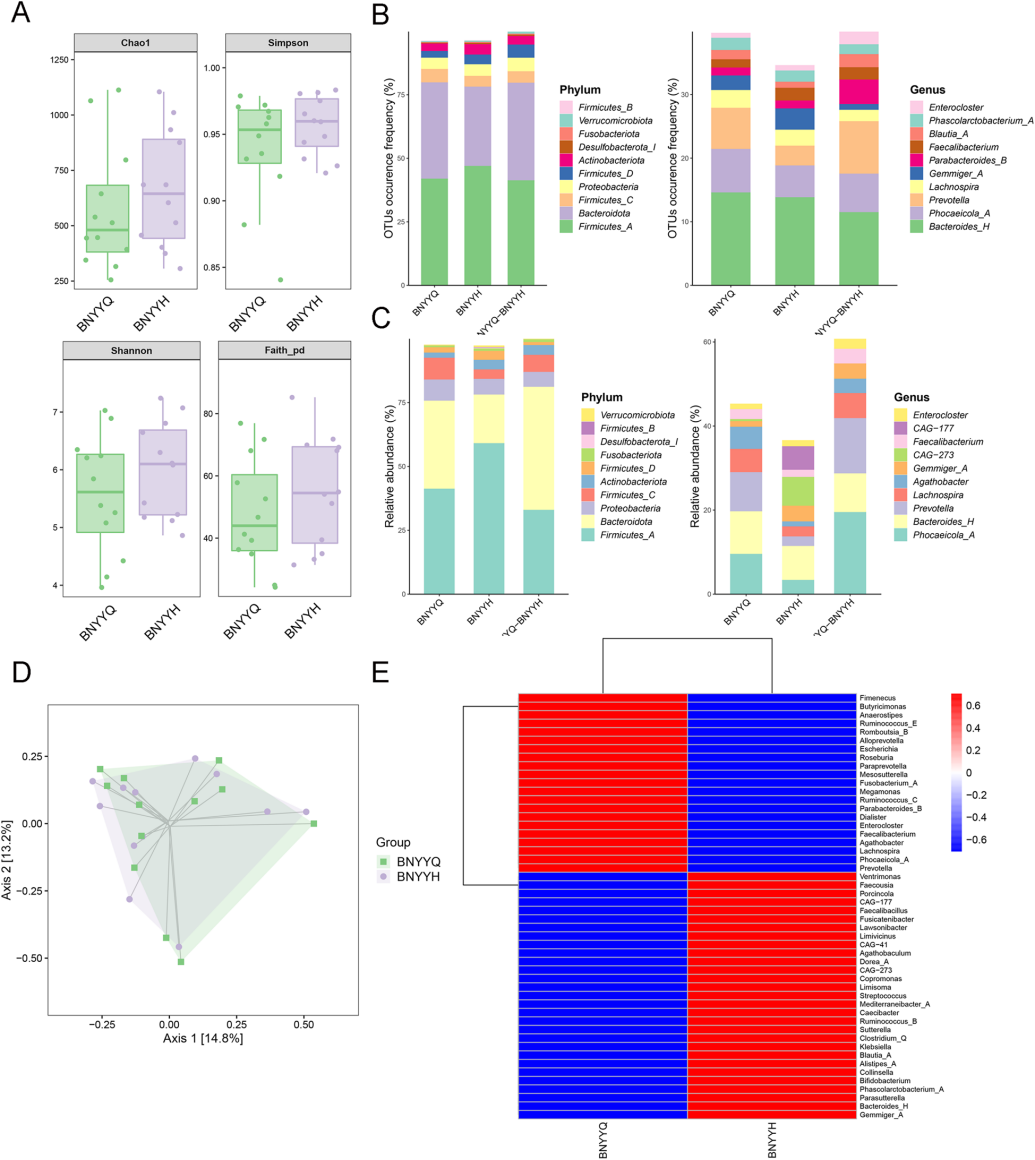
Changes in the gut microbiota of patients with obesity before (BNYYQ) and after (BNYYH) treatment with beinaglutide. **A** α-Diversity of the gut microbiota (Chao1, Simpson, Shannon, and Faith PD indices). **B** Abundance of the corresponding amplicon sequence variant (ASV)/operational taxonomic unit (OTU) in each region was counted at the phylum and genus level. **C** Number of corresponding ASVs/OTUs per region was counted at the phylum and genus level. **D** β-Diversity of the gut microbiota. **E** Heat map analysis of differences in intestinal flora between healthy individuals and patients with obesity

Firmicutes, Bacteroidota, and Proteobacteria remained the most abundant phyla after weight loss in patients with obesity, with a significant increase in Firmicutes abundance (Fig. 5B, C). α- and β-Diversity analyses were performed to determine whether beinaglutide altered gut microbiota diversity, revealing differences in the Chao1 and Faith PD indices (Fig. 5A, D). At the genus level, the relative abundances of *Prevotella*, *Lachnospira*, *Dialister*, *Anaerostipes*, and *Parabacteroides_B* decreased, whereas those of *Lawsonibacter*, *Collinsella*, *Gemmiger_A*, *Blautia A*, *Phascolarctobacterium A*, and *Bifidobacterium* increased after weight loss in patients with obesity (Fig. 5E).

### Adverse drug reactions

In this study, 9 people experienced mild-to-moderate nausea and abdominal distension and 1 person experienced headache after the use of Beinaglutide. The incidence of adverse drug reactions was 30.3%, which was mainly concentrated during meals and Beinaglutide dosage, and all of the above symptoms could be relieved 2–3 h after the appearance of the symptoms, and there were no events, such as acute pancreatitis and hypoglycemia.

## Discussion

### Main interpretation

GLP-1 is an enteric insulinotropic hormone secreted and produced by intestinal L-cells, which increases insulin secretion and inhibits glucagon secretion in a glucose-dependent manner. GLP-1RAs suppress appetite, decrease gastric emptying, and increase energy expenditure via the gut–brain axis to achieve weight loss, especially the reduction of visceral fat [13, 14]. Postprandial GLP-1 secretion levels are significantly lower in patients with obesity than in healthy individuals [15]. Beinaglutide, a short-acting GLP-1RA with 100% homology to human GLP-1 [6], was approved by the State Drug Administration of China for glycaemic control in adults with type 2 diabetes or adult weight management. Pre-meal use of beinaglutide increases postprandial GLP-1 levels and can effectively reduce waist circumference, body weight, and fat mass in patients classified as overweight and obese [6, 16].

In this study, a sustained significant reduction in visceral fat area over baseline levels was observed in patients with obesity who received beinaglutide treatment for 12 weeks. This was accompanied by significant decreases in BMI, body fat, body fat percentage, TC, LDL-C, and inflammatory markers, consistent with significant increases in insulin resistance, all of which correspond with the results of previous studies [7, 16, 17]. Increased visceral fat area is often accompanied by insulin resistance and systemic chronic inflammation [18, 19]. Beinaglutide treatment significantly reduces the visceral fat area and effectively improves insulin resistance, lowering the risk of hyperlipidaemia, diabetes, and cardiovascular disease [20, 21]. In contrast to previous studies, HDL-C levels were found to decrease after 4 weeks of treatment and increase after 12 weeks of treatment, which may be attributed to the mechanism of the effect of GLP-RAs on lipids is not clear. HDL-C synthesis is mainly affected by various factors, such as ApoA-I, lecithin cholesterol acyltransferase and etc. Short-term changes of the above mentioned substances affect the synthesis of HDL-C, Overall evaluation GLP-1RAs have a small effect on HLD-C synthesis, transport and metabolism [22]. A significant decrease in FPG was observed after 12 weeks of beinaglutide treatment, which was attributed to weight loss and a significant improvement in insulin resistance [6, 16].

We collected faecal specimens from healthy participants and participants with obesity for *16S* rRNA amplicon sequencing before and 12 weeks after beinaglutide treatment. In both healthy individuals and patients before and after beinaglutide treatment, three specific phyla were consistently dominant: Firmicutes, Bacteroidetes, and Proteobacteria. α- and β-Diversity analyses were performed to determine whether beinaglutide altered gut microbiota diversity, revealing differences in the Chao1 and Faith PD indices. This indicates that the gut microbiota of patients with obesity exhibited lesser abundance and diversity than that of the healthy population. Importantly, beinaglutide treatment restored the α-diversity of gut microbiota to a certain extent. Beta diversity analysis using principal coordinates showed no significant difference in beta diversity of intestinal flora between patients with obesity and healthy individuals, and before and after beinaglutide use in patients with obesity. Analysis of subordinate levels by heat map revealed that patients with obesity were relatively deficient in anti-obesity genera compared to healthy individuals. *Anaerostipes*, *Parasutterella*, *Megamonas*, *Dialister*, *Lachnospira*, and *Prevotella * increased in relative abundance; *Lawsonibacter*, *Roseburia*, *Collinsella*, *Blautia_A*, *Phascolarctobacterium A*, *Bifidobacterium* and *Feacalibacterium* decreased in relative abundance. After 12 weeks of treatment with Beinaglutide, decreased relative abundance of *Prevotella*, *Lachnospira*, *Dialister*, *Anaerostipes*, and *Parabacteroides_B* while increased relative abundance of *Lawsonibacter*, *Collinsella*, *Gemmiger A*, *Blautia A*, *Phascolarctobacterium A*, and *Bifidobacterium* in patients with obesity. The findings are consistent with those of Pedersen et al*.*, who reported that the abundance of *Prevotella* was positively correlated with obesity, blood glucose, and insulin levels [23]. Moreover, another study found that overweight adults with a high abundance of *Prevotella* lost more weight than participants with a low abundance [24]. It is possible that the abundance of *Prevotella* among gut microbiota plays a key role in the production of different types of short-chain fatty acids (SCFAs), thus affecting appetite and body weight [25]. *Lachnospira* are more abundant in individuals with obesity [26]. Duan et al. reported that semaglutide reduced the relative abundance of *Lachnospira,* which contributed to a decrease in body weight in obese mice [27] and also interfered with SCFA production to control body weight [28]. In contrast, *Gemmiger A* was more abundant in the gut flora of lean animals [29], being negatively correlated with SCFA production, particularly with that of acetic acid [30]. *Blautia A* is a gut flora negatively correlated with visceral fat area [31]. Insulin sensitivity correlates with higher abundance of *Phascolarctobacterium* and lower abundance of *Dialister* [32]. This study also supported that the relative abundance of *Prevotella*, *Lachnospira*, and *Dialister* decreased in patients with obesity after weight loss, while the relative abundance of *Blautia A* and *Gemmiger A* increased. SCFAs upregulate the expression of adipose tissue thermogenesis-related genes peroxisome proliferator-activated receptor-γ (PPARγ) coactivator 1 α and uncoupling protein 1 (UCP1) [33, 34] as well as enhance the expression of adipose tissue thermogenesis-related proteins, such as acetyl coenzyme a oxidase, carnitine palmitoyltransferase I, and uncoupling protein 2 (UCP2), to stimulate adipose tissue thermogenesis and energy expenditure, ultimately alleviating obesity [35, 36].

### Limitations

This study has certain limitations. The sample size was relatively small, and this analysis only partially characterises the effect of beinaglutide on the gut microbiota of patients with obesity. In addition, the study did not evaluate the effect of varying beinaglutide doses. Thus, we could not assess the dose dependency of the observed changes. Further studies with larger and more diverse cohorts are required to validate our findings. Owing to the extreme complexity of gut flora–host interactions, as well as our knowledge and research capacity, the potential mechanisms underlying such interactions were not explored further, including the relationship between gut microbiota and SCFAs, as well as the mechanisms underlying the effect of specific microbiota on SCFAs. In the future, the molecular mechanisms utilised by the microbiota should be explored through the collection of more samples for macrogenomic sequencing. To fully elucidate the capacity for modifying gut microbiota for obesity prevention and treatment, large-scale intervention studies that distinguish correlation from causation are also required.

### Conclusion

This study showed that beinaglutide can be used as a treatment to effectively reduce body weight, visceral fat area, and alter the gut microbiota composition. More specifically, beinaglutide restored the richness and diversity of the gut flora in patients with obesity, decreasing the relative abundance of *Prevotella*, *Lachnospira*, and *Dialister* while increasing that of *Blautia A and Gemmiger A*. However, more in-depth analyses are required to determine the extent to which beinaglutide modulates the gut microbiota achieve weight loss.

## Supplementary Information

Supplementary Material 1.

Supplementary Material 2.

Supplementary Material 3.

Supplementary Material 4.

Supplementary Material 5.

Supplementary Material 6.

## Data Availability

The datasets generated during and analyzed during the current study are not publicly available but are available from the corresponding author on reasonable request.

## Abbreviations

WC: Waist circumference
BMI: Body mass index
HR: Heart rate
SBP: Systolic blood pressure
DBP: Diastolic blood pressure
TC: Total cholesterol
HDL-C: High density lipoprotein-cholesterol
LDL-C: Low-density lipoprotein cholesterol
TG: Triglyceride
ALT: Alanine aminotransferase
AST: Aspartate aminotransferase
GGT: γ-Glutamyl-transpeptidase
ALP: Alkaline phosphatase
UA: Uric acid
BUN: Blood urea nitrogen
Cr: Creatinine
eGFR: Estimated glomerular filtration rate
ADPN: Adiponectin
HOMA-IR: Homeostasis model assessment of insulin resistance
FPG: Fasting plasma glucose
HbA1c: Glycated haemoglobin A1c
VFA: Visceral fat area
BMR: Basal metabolic rate
NAFLD: Non-alcoholic fatty liver disease
