# Supplementary figures and images for: Effects of beinaglutide on visceral fat area and gut microbiota in obesity
Source: Eur J Med Res. 2025 Jun 4;30:448. doi: 10.1186/s40001-025-02585-5 (PMC12135259; doi:10.1186/s40001-025-02585-5)

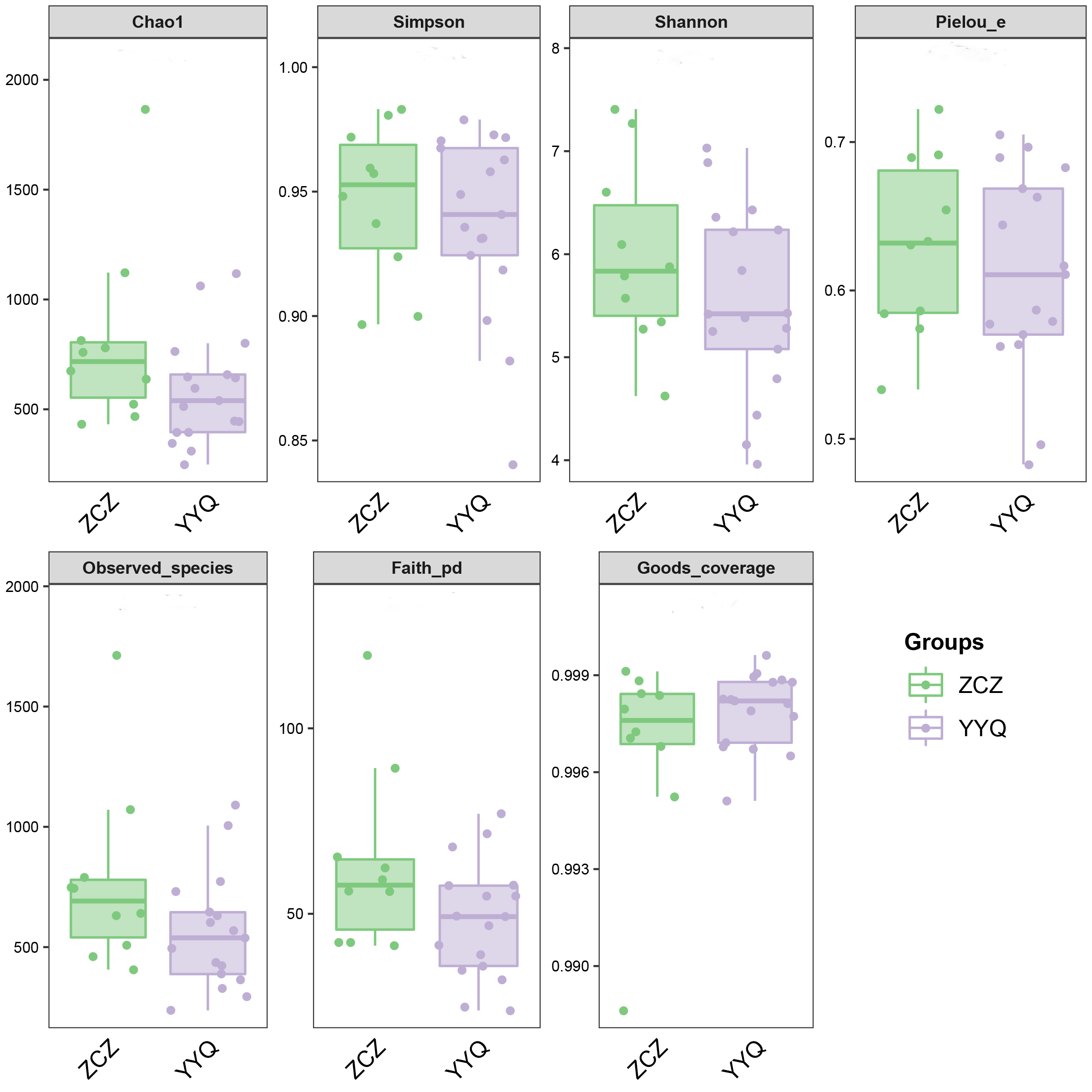

Supplement: Supplementary file 1 — Supplementary Material 1. [file 40001_2025_2585_MOESM1_ESM.png]

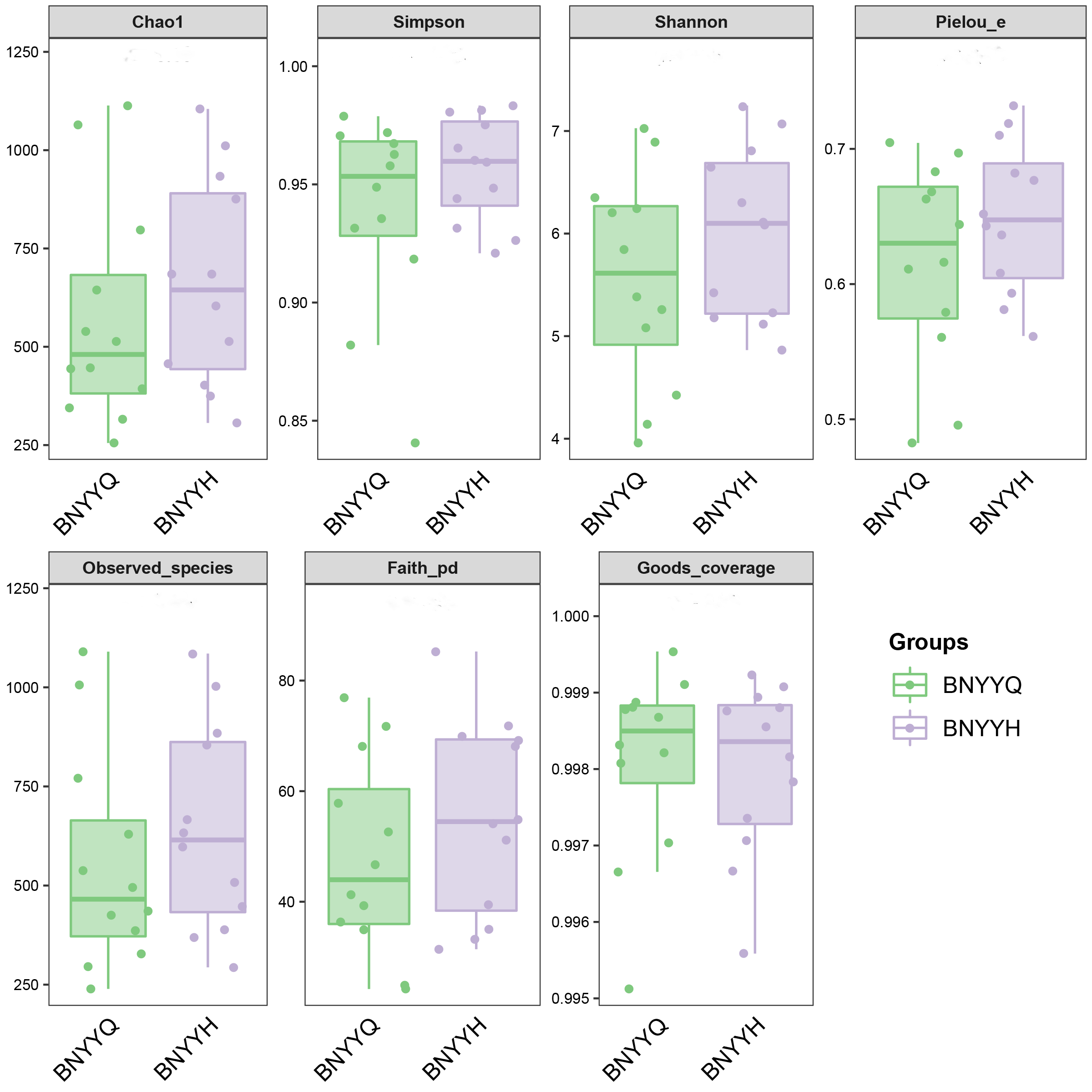

Supplement: Supplementary file 2 — Supplementary Material 2. [file 40001_2025_2585_MOESM2_ESM.png]

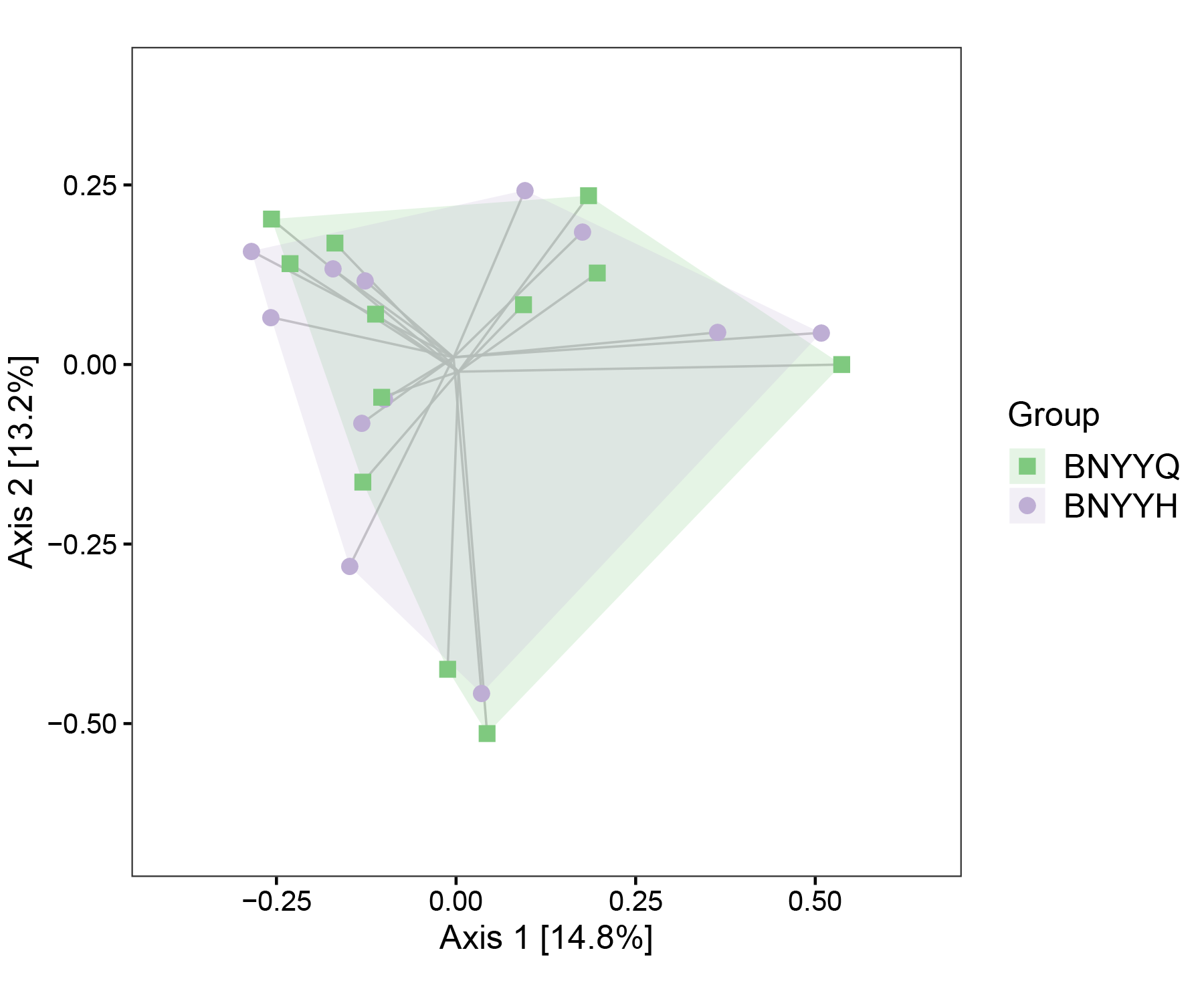

Supplement: Supplementary file 3 — Supplementary Material 3. [file 40001_2025_2585_MOESM3_ESM.png]

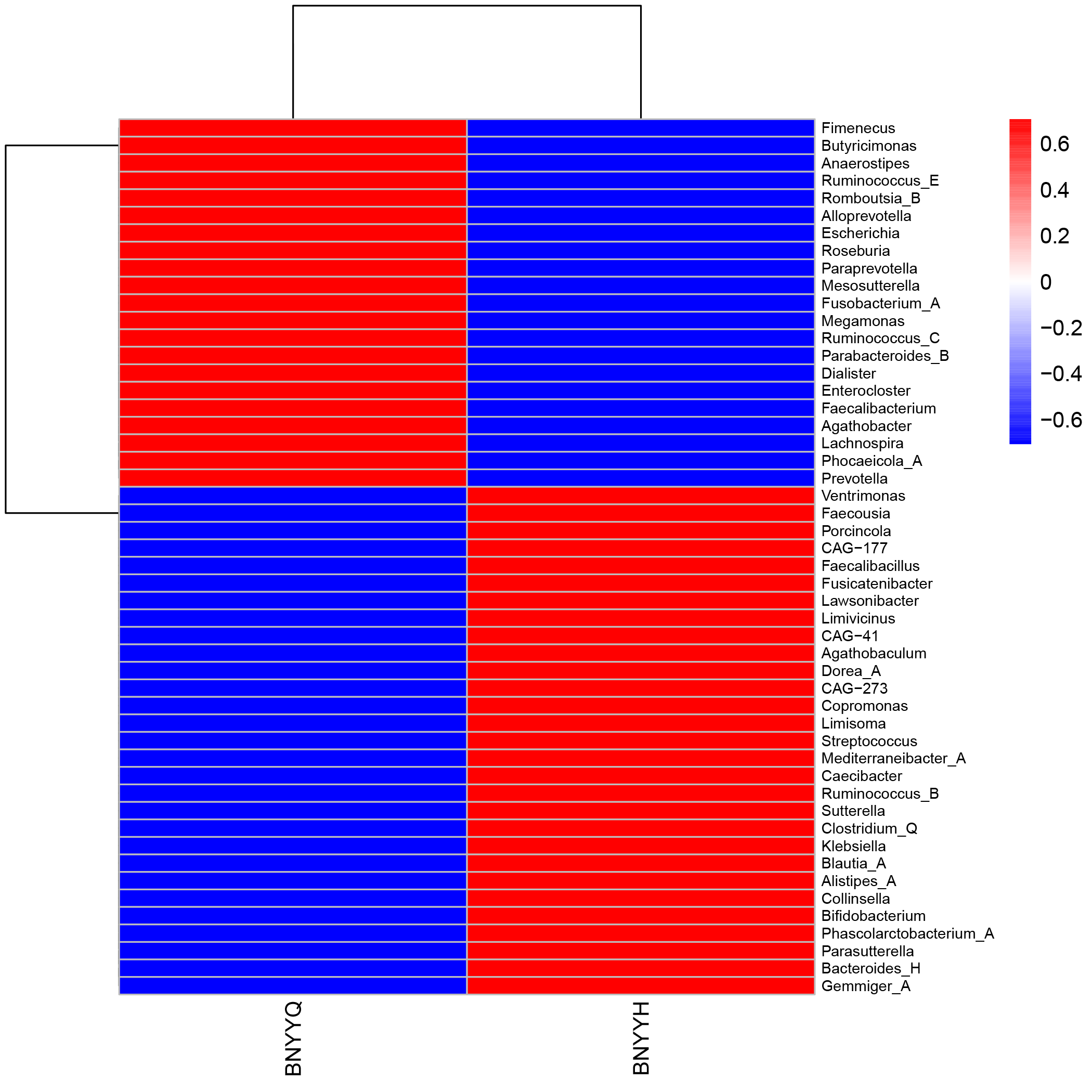

Supplement: Supplementary file 4 — Supplementary Material 4. [file 40001_2025_2585_MOESM4_ESM.png]

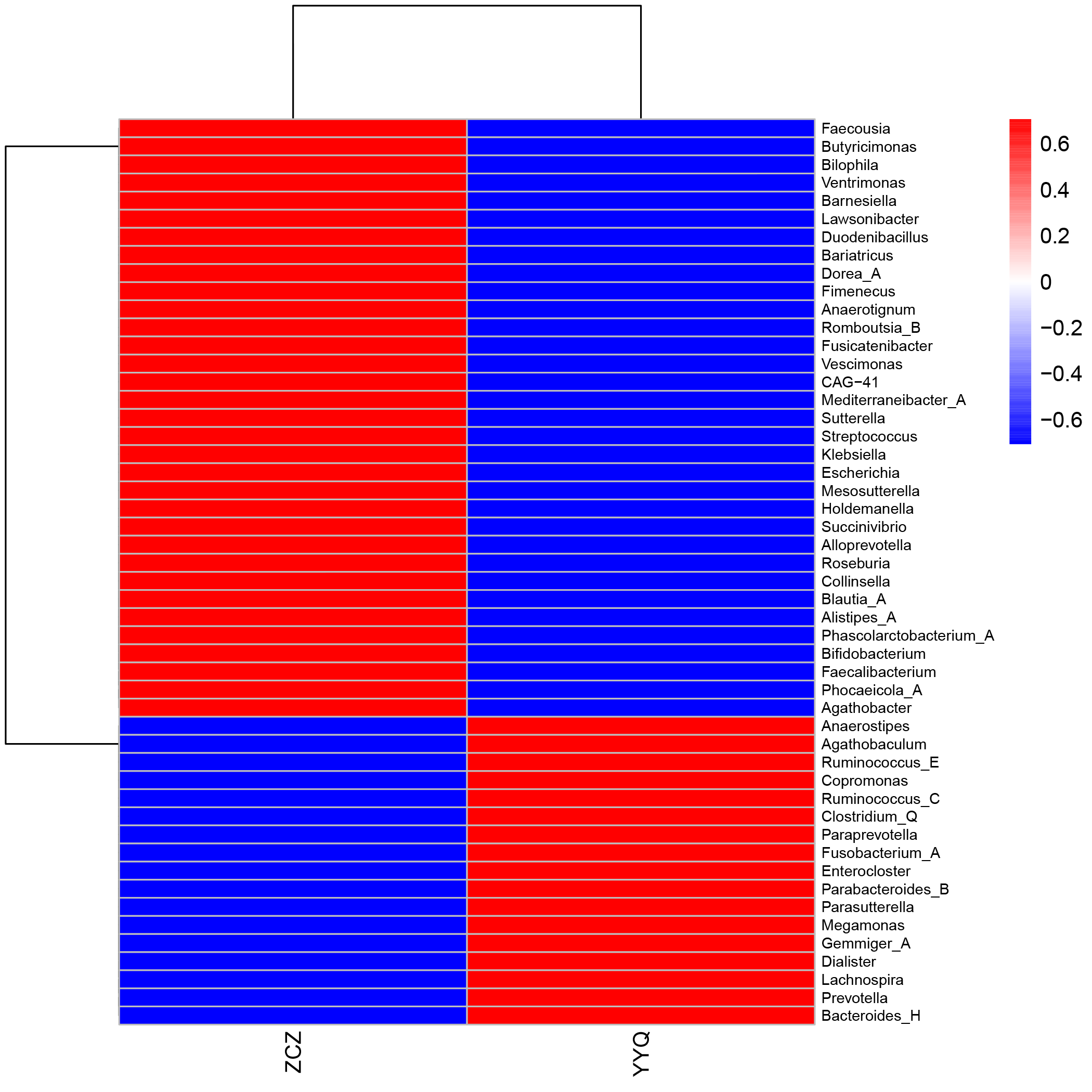

Supplement: Supplementary file 5 — Supplementary Material 5. [file 40001_2025_2585_MOESM5_ESM.png]

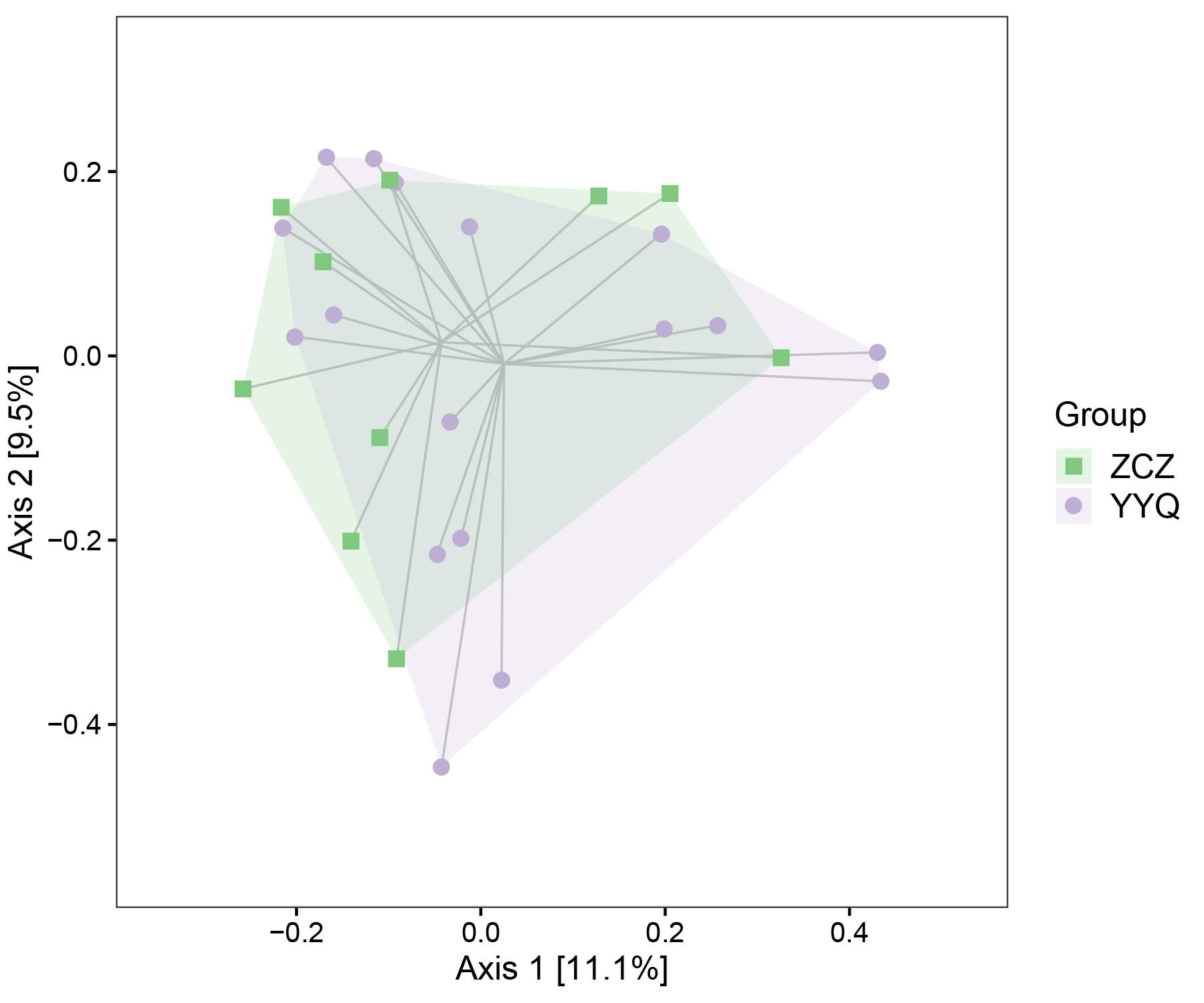

Supplement: Supplementary file 6 — Supplementary Material 6. [file 40001_2025_2585_MOESM6_ESM.png]
